# A qualitative interview study to investigate opportunities for improvement in routine asthma care using a clinical decision support system

**DOI:** 10.1038/s41598-025-25438-x

**Published:** 2025-11-24

**Authors:** Holly Tibble, Jaime Garcia Iglesias, Alexandria Chung

**Affiliations:** 1 Usher Institute, The University of Edinburgh, Edinburgh, UK; 2 Botswana Sexual and Reproductive Health Initiative, Botswana Harvard Health Partnership, Princess Marina Hospital, Gaborone, Botswana

**Keywords:** Asthma, CDSS, Interview, Software, Codesign, Public health, Health services

## Abstract

More than 8 million people in the UK have been diagnosed with asthma, a chronic respiratory condition which leads to the death of more than 25 people per week. Clinical decision support software (CDSS) can be used to improve patient care by improving clinical accuracy or by increasing the efficiency of clinical practice. This study aimed to identify desired functionalities of a software tool for asthma care, from both primary and specialist health care providers. Qualitative data were collected from semi-structured interviews with 19 participants. Eight functionalities were identified for prioritisation for the development of clinical decision support system in asthma, including diagnosis support, medical history retrieval, and adherence to best practice guidelines. Parallels were drawn with successful CDSS applications in other medical fields, such as radiology, highlighting the value of interdisciplinary learning and adaptation. Prioritising user-centred design, and aligning processes with existing workflows, may allow software tools to lighten administrative burdens, promote proactive patient care, and improve patient outcomes. Healthcare software development must focus on creating tools which are intuitive, reliable, and co-designed, in order to increase uptake and sustained impact.

## Introduction

Asthma affects 300million + people worldwide, including 11.6% of children aged 6–7 years^1^. In the UK alone more than 8 million people (12%) have been diagnosed with asthma, with 160,000 new cases recorded annually^1^. Asthma accounts for 2–3% of primary care consultations, 60,000 hospital admissions and 200,000 bed days/year in the UK^2^. Asthma also leads to the death of more than 25 people per week on average in the UK^3,4^—half of whom die before calling for, or receiving, any emergency care^5,6^.

Clinical Decision Support Systems (CDSSs) are software tools used in routine clinical practice to assist medical decision making through either predicting the risk of outcomes under specific conditions, or through processing data to allow the user to make the decision more efficiently and optimally^7,8^. When predicting outcomes, they can be used as a second expert opinion for a healthcare provider (HCP). As the number of patients in the data used to train a CDSS will typically far exceed the number of patients seen during the career of any individual HCP, CDSSs may be able to better draw on knowledge of unusual cases such as patient with rare comorbid diseases^9^. They may also be able to support staff members in their learning^10^, and provide balance to certain human (cognitive and/or cultural) biases^11,12^. This may be particularly pertinent as pressure on the UK primary care system continues to grow^13–15^, increasing our reliance on imperfect heuristic decision making processes^11^.

There has been a substantial increase in development of CDSSs for asthma in recent years^16–19^. This has resulted in a number of successful trials in clinical decision support tools for asthma care, including in supporting the interpretation of spirometry test results^20,21^, flagging high-risk patients in primary care^22,23^, identifying those overdue for influenza vaccinations^24^, and improving quality of care by supporting patient adherence to evidence-based guidelines^22,25–28^. Despite these promising results, to the extent of our knowledge, the only system currently operational with the UK is the London Asthma Decision Support Tool (LADS)^29^: created through a collaboration between North West London and South East London Integrated Care Boards, and covering 80 primary care networks, which aims to better identify high risk asthma patients and provide population level information about the condition.

A 2023 Canadian study identified several key recommendations for integrating asthma tools and clinical guidelines into primary care records, including designing tools to achieve specific outcomes, prioritising the end user in the design process, and incorporating tools into existing workflows^30^. Guided by these principles, the objective of this study was to consult with HCPs in both primary and specialist care to ascertain the components of standard, routine asthma care which could benefit from computer-aided decision support, and the specific functionalities required for success.

## Methods

### Study design

This qualitative study comprises interviews from HCPs to gain deep insights into how real-time data-driven insights could be used in asthma management. Qualitative data were collected from semi-structured interviews, providing rich and nuanced data. Participants were all asked to describe their current standard care, and then follow-up questions were asked to explore how various routine tasks could be made more efficient or beneficial to patients with the support of computer aided decision support.

All methods were approved by the Edinburgh Medical School Research Ethics Committee, and were carried out in accordance with their guidelines. Informed consent was obtained from all participants.

### Study population

We aimed to recruit 16–25 HCPs for a one-off interview, lasting 30–60 min, through convenience sampling. Recruitment was split between primary care (general practitioners, primary care practice pharmacists, and asthma nurses), and secondary care respiratory specialists (hospital respiratory specialists and respiratory physiologists), with the aim to recruit at least 8 participants from each setting. The inclusion criteria were:

- Participant is willing and able to give informed written consent for participation in the study,
- Participant has been a practising UK HCP for at least one year,
- Participant has had consultation with at least one patient with confirmed or suspected asthma in the past six months,
- Participant speaks English fluently.

### Recruitment process

Recruitment took a multi-pronged approach; the study was advertised on social media (LinkedIn and X, formerly Twitter) and mailing lists, as well as through the researcher’s own networks. Interested individuals were provided with the Participant Information Sheet and the Informed Consent form.

When consent to participate was provided, individuals were sent a link to an online screening form. This screening form requested their name (for linkage to the consent form), confirmation of their eligibility, the outward section of their postcode, their age group, gender, and ethnicity.

Once screening was completed, participants were invited to an online interview. Participants were free to withdraw from the study at any stage, in which case their screening data and interview transcript were removed.

### Data collection, synthesis and analysis

We conducted in-depth semi-structured interviews to explore tasks and processes in routine asthma care which might benefit from digital support. The interview guide was co-written by all authors. At the start, the interviewer (HT) introduced herself, her background, and the primary motivation for conducting this research. The first questions allowed the participant to detail their role and typical routines, and then the questioning transitioned towards their wants and needs to improve their performance.

All interviews were conducted by HT on Microsoft Teams, a video-conferencing platform. Her background is as a data scientist and epidemiologist (early career researcher, PhD), focussing on respiratory health. She maintained a reflexive blog throughout the process to reflect on possible personal biases during interviewing or coding, which was discussed with the study team during meetings. The three key principles which guided the interview approach were humility (no-one knows everything), compassion (healthcare providers are under heavy burden), and acceptance (the interviewer and interviewee have different experiences and priorities). These principles were used as a framework for starting each reflective blog. Interview duration ranged from 15 to 59 (mean 32, SD 11) minutes, with variations due to the complexity of roles and the number of suggested CDSS functions. They were then transcribed, using the Teams auto-transcription as a baseline, and manually reviewing the recorded video files to make edits where required.

Qualitative data was coded inductively to identify initial topics, manually in Microsoft Word and Microsoft Excel. Interviews were conducted until reaching data saturation. This was established when no new perspectives were identified relative to previously obtained data.

Emerging themes were discussed with co-investigators with qualitative research expertise (JGI) and clinical experience (AC), as well as through peer debriefing with a wider network of qualitative researchers within the institute (Usher Institute, University of Edinburgh) during an internal ‘work in progress’ seminar series. A comprehensive and transparent record was maintained throughout, to form an audit trail detailing the addition of new codes, and the collapse of individual codes into a single entity.

Thematic mapping was then conducted, and recorded in detail, to organise topics into groups based on common functionality. Transcripts were revisited to confirm the placement of individual topics and the consistency of the groups.

## Results

### Recruitment

Recruitment formally commenced on July 4th, 2024. 19 participants were recruited, of which 8 were based predominantly in primary care (4 GPs [General Practitioners] and GP registrars, 2 practice nurses, and 2 practice pharmacists) and 11 were based in secondary or tertiary care, or a mixed care setting (Table 1). The latter group encompassed a wide variety of clinical roles including respiratory consultants, community respiratory physiotherapists, asthma nurses, and paediatricians with specialist interest in asthma and allergy (SPIN).

**Table 1:** Characteristics of participants.

Characteristic	Participants (Percentage)
Age Group	
35 and under	5 (26%)
36 to 50	8 (42%)
51 and over	6 (32%)
Gender	
Female	12 (63%)
Male	7 (37%)
Ethnicity	
White	13 (68%)
Asian	4 (21%)
Black	1 (5%)
Other	1 (5%)
Years spent in clinical role	
Up to 5 years	3 (16%)
6 to 10 years	3 (16%)
11 to 20 years	6 (32%)
21 to 30 years	6 (32%)
Longer than 30 years	1 (5%)
Weekly hours in clinical setting	
Less than 20	7 (37%)
21 to 30	3 (16%)
31 to 40	6 (32%)
41 to 48 (medical trainee band 1a-c or equivalent)	2 (11%)
49 to 56 (medical trainee band 2 or equivalent)	1 (5%)
Healthcare setting	
Primary	8 (42%)
Secondary and Tertiary (or mixed)	11 (58%)
Country or region of work	
Northern Ireland	2 (11%)
Wales	1 (5%)
Scotland	6 (32%)
London	3 (16%)
Other England	7 (37%)

### Overview of functionalities

The healthcare providers interviewed suggested many potential functionalities in a CDSS that could lead to improvements, which have been broadly grouped into functions to improve clinical accuracy or to increase consultation efficiency. The topics are briefly summarised in Table 2, and described in more detail in the following sections.

**Table 2:** Summary of potential functionalities.

Objective	Functionalities	Opportunity
Improve clinical accuracy	Diagnosis	Support the diagnosis of asthma
	Best-practice refreshers	Tool to remind healthcare providers of the latest best practice guidelines
	Selection and optimisation of maintenance treatment	Tool to support asthma prescribing
	Risk Categorisation	Tool to support estimating risk of patient outcomes
Increase consultation efficiency	Clinical Admin	Tool to support administrative tasks in healthcare provision
	Medical History Retrieval	Tool to support retrieval of pertinent information from historical medical records
	Support with interpretation of tests	Tool to support with the interpretation of medical test results
	Translation of tools from other chronic diseases	Existing tools used in management of other chronic diseases being repurposed for asthma

### Functions to improve clinical accuracy

#### Diagnosis

Several participants highlighted the accurate diagnosis of asthma in primary care as a fundamental but often difficult task, particularly in light of changing and subjective guidelines, the diversity of presentations of asthma symptoms, and the multitude of similar respiratory conditions.

There isn’t a hard and fast diagnosis based on set criteria that if you’ve got these criteria, then you’ve got asthma. And if you don’t have them, you don’t. It’s a whole bunch of history examination and investigation findings that would push you more or less in the direction of asthma.

Participant 5: Secondary Care Respiratory Specialist with 11–20 years of experience

#### Best practice refreshers

Many participants commented on the difficulty of remembering best practices in asthma care, and how having a tool to prompt possible next steps may be useful to ensure that the best care is offered, without increasing their cognitive load.

I’ve got so much to remember on a daily basis, so many patients coming to see me that actually… we’re only human. We can’t remember everything with every consultation and we will make mistakes at some point. So to have a computer aided system that’s going to support me in delivering the best care I can to patients is perfect.

Participant 16: Primary Care Practice Nurse with 11–20 years of experience

It was often remarked by participants in secondary and tertiary care that there were best practices not being adhered to in primary care, which resulted in worse outcomes by the time they were seen by specialists.

You could have a practice nurse with very little experience and hardly any training. They’ve got this ARRS thing [the Additional Roles Reimbursement Scheme] - their replacement role scheme. They got lots of money from NHS England to pay for pharmacists and other allied healthcare professionals to work in GP practices, doing asthma and COPD [chronic obstructive pulmonary disease] reviews with minimal training.

Participant 12: Respiratory Nurse Specialist and Clinical Practice Educator with 1–5 years of experience

There was actionable information [in a patient’s records], for such a long time that no one was looking at. It’s really not an uncommon thing. But things like that happen and then by the time they get to you, you think, ‘well, yes, you have asthma, but now you’ve got so many other problems as well.’

Participant 8: Secondary Care Respiratory Specialist with 11–20 years of experience

Several primary care providers also commented that, by the nature of their roles, they are required to cover such a wide variety of health concerns, that staying abreast of changing guidelines can be a mammoth task.

Because you are a generalist, so it can be hard to keep up to date with all the knowledge.

Participant 1: GP Registrar with 6–10 years of experience

I think anyone could forget to cover an area, particularly when they’re bringing in other problems or other issues. You’ve got your agenda. They’ve got theirs. And there must be areas where I’m not following guidelines because how do we in general practise, how do we know the most up to date about everything?

Participant 11: Primary Care Practice Nurse with 21–30 years of experience

The most commonly mentioned area for improvement in primary care was reducing the amount of short-acting beta-2 agonist (SABA) reliever inhalers being prescribed. SABA inhalers are often prescribed to relieve exacerbations. However, there is high prevalence of individuals using SABA inhalers at a rate that would indicate that their maintenance therapy was not sufficient to achieve symptom control. High SABA use has been found to be associated higher risk of adverse outcomes, including mortality^31,32^.

I don’t ever call it SABA overuse. I call it SABA over prescribing because SABA over use very much seems to be it’s patient’s problem. It’s their fault, it’s them that’s doing it when of course really the problem is with the prescribers are prescribing it for them.

Participant 17: Integrated Care Specialist Nurse and Clinical Lead with over 30 years of experience

#### Selection and optimisation of maintenance treatment

As well as the over-prescribing of SABAs, there were common concerns about prescribing the right asthma controller inhalers.

There are so many different ones now that we’re a bit de-skilled in deciding. … Which would benefit my patient in front of me best?

Participant 2: GP with 21–30 years of experience

Some participants highlighted the value in a tool which could prompt the next step if a patient was not responding well to their current therapy, as well as support for when a patient could safely be stepped down to a lower level. The latter was also included in the 2024 joint guidelines for the diagnosis and management of asthma, were written by BTS, SIGN, and NICE (NG245^33^, as one of their eight key recommendations for research. Stepping down asthma therapy when an individual is well controlled can reduce the risk of side effects and reduce excess medication costs. Additionally, higher intensity regimens often comprise multiple individual medications. These require more time to use, and may have different techniques for each. Stepping down therapy therefore may increase patient satisfaction. Ultimately, the best dose of inhaled corticosteroids (ICS) is the lowest dose that keeps the patient free of symptoms.

#### Risk categorisation

Categorisation of risk was a common feature discussed, although clinicians described the potential usefulness of CDSSs in various ways such as: aiding in decision-making about short-term risk; prioritising patients in primary care; determining referrals to specialist care; and support the understanding and interpretation of compound risks.

The first potential use was to aid decision making about short term risk, including whether a patient could be safely discharged from secondary care, and whether a patient should be directed from primary to emergency care.

Now if I give them a respiratory rate and saturation, what is their current risk? … Should they be monitored in practise or should they go up to A&E for example?

Participant 2: GP with 21–30 years of experience

One participant particularly noted that there was inconsistency between healthcare providers in the conditions that would lead them to feel they could safely discharge a patient.

We haven’t actually discussed this as a team… I think everyone’s probably got different barometers.

Participant 10: Secondary Care Respiratory Specialist with 11–20 years of experience

The second use was in prioritising patients in primary care, particularly in light of the burden on health services.

Identify the patients who you need to see first and forget the ones that you don’t need to see because you’re not going to see 100% anyway.

Participant 17: Integrated Care Specialist Nurse and Clinical Lead with over 30 years of experience

The third use was for determining who should be referred to specialist care from primary care, including those who are eligible for biologic therapy. Biologic therapy uses synthetics antibodies to stop the processes that cause inflammation in the lungs. There are currently six biologic treatments approved to treat severe asthma in the UK, however due to their high expense there are strict criteria for who can access them.

And then at the end of it, ‘this patient may be eligible for biologics’ and perhaps even flagging up which biologics they might be eligible for.

Participant 4: Secondary Care Respiratory Specialist with 21–30 years of experience

A common concern from tertiary care providers was that they saw many patients in their clinics where poor prescribing and/or poor adherence were clearly the root cause of their symptoms—issues which they felt should have been dealt with in primary care.

The worst scenario is when they haven’t really looked properly at the asthma symptoms even, they haven’t looked at adherence, and it’s quite frustrating to see a patient whose asthma control is poor because they’re just not picking up their ICS. And that obviously should be dealt with in primary care.

Participant 4: Secondary Care Respiratory Specialist with 21–30 years of experience

On the flip side, there were also concerns that some extremely at-risk patients were not being referred, when they would have likely greatly benefitted from biologic therapy.

From the point of biologic treatment, we’re obviously not seeing the amount of people that we should be seeing and it’s only the tip of the iceberg. And I think if there’s an easier way to for primary care to be able to pick out those patients….

Participant 13: Secondary Care Asthma Nurse with 21–30 years of experience

A fourth use was to support the understanding and interpretation of compound risks—characteristics which might together affect the likelihood of diagnosis or risk of poor outcomes more or less than each characteristic in isolation.

I know that you having a family history of asthma, that makes you in some ways more likely for that to be asthma, I don’t really know if you’ve got this, this, this and this all together, if that makes you 95% more likely to have asthma than just one of them? … If they’ve got this other thing or this protective thing, it gets quite complicated … [it’s] very hard to study unless you have a lot of data and these sort of machine learning algorithms to process them.

Participant 10: Secondary Care Respiratory Specialist with 11–20 years of experience

### Functions to increase consultation accuracy

#### Clinical admin

Some participants highlighted inefficient and ‘tedious’ administrative tasks, which they felt could be more resourcefully handled by semi-automatic software. These included writing letters to primary care from secondary and tertiary care departments:

Say your conversation was recorded, and then it was transcribed into a letter, and then you just maybe edited a little and that would certainly be less time consuming.

Participant 13: Secondary Care Asthma Nurse with 21–30 years of experience

#### Medical history retrieval

Almost all participants described tasks of reviewing previous medical records, including primary care consultation records, and prescribing and dispensing records as often laborious.

It’s a lot of detective work. And it’s a lot of investment and time and quite often that has to happen before the clinic.

Participant 9: Paediatric trainee doctor with a specialist interest in asthma and allergy (SPIN), with 11–20 years of experience

When such administrative tasks needed to take place during a consultation, participant healthcare providers feared patients might regard this as lower quality care.

People often complain, you go and see your GP and they’re just staring at the screen. They’re trying to find everything you know, they’ve got only got 10 min. They might have only just finished a patient, got everything on their mind, and you’ve got to jump into another patient.

Participant 12: Respiratory Nurse Specialist and Clinical Practice Educator with 1–5 years of experience

People highlighted the opportunity for streamlining this kind of work in several different ways, including automatically pulling information on request from past consultations and letters, and creating summary care records.

And I think one of the benefits has to be that those things should be being able to be done for you automatically - like scrolling through endless appointment letters to try and pull that information. I think there’s definitely, ways that it could all be pulled and tied up in a nice way, it would save you so much time and hassle and stress, but I also think there’s an element that when you’re scrolling by eye in a busy clinic and you’re in a rush, you definitely could miss things as well.

Participant 9: Paediatric trainee doctor with a specialist interest in asthma and allergy (SPIN), with 11–20 years of experience

It would be great for GPs and secondary care physicians to have a sort of streamlined way where all that information just gets sort of put in a standardised template and you know goes through secondary care.

Participant 4: Secondary Care Respiratory Specialist with 21–30 years of experience

#### Support with interpretation of tests

Several participants mentioned a desire for support with the interpretation of tests, in particular for lung function.

Currently the hospital’s doing [spirometry tests] but doesn’t want to interpret them all. And then so you attempt to interpret it. But actually, I reckon AI could do that better and then have a clinician oversee it. I believe it’s doing X-rays and things.

Participant 11: Primary Care Practice Nurse with 21–30 years of experience

One participant specifically remarked on wanting tools which helped them to explain the results to their patients.

I am a big believer in trying to explain what what’s actually the problem. … Spirometry loops and things are a bit harder to… kind of quite abstract. Squiggles which don’t necessarily mean a lot. The numbers don’t mean a lot unless you’ve been trained to interpret them or read them. … I’d like to be able to show those to patients and explain them properly.

Participant 8: Secondary Care Respiratory Specialist with 11–20 years of experience

#### Translation of tools from other chronic diseases

Several participants drew parallels between standards of care in asthma and other diseases: heart disease, COPD, atrial fibrillation, and most prevalently, diabetes.

What’s happened this year? We use that a lot for cholesterol, diabetes, things like that. Trends. We don’t use that so much for asthma to be honest.

Participant 18: Primary Care Pharmacist with 6–10 years of experience

So you put in all their demographics and whether they smoke or not, it comes up with a 10 year heart disease risk. And then if you say change them to a non-smoker, it reduces their heart disease risk. So you could do that with asthma, couldn’t you?

Participant 2: GP with 21–30 years of experience

Whoever is seeing and reviewing the asthma at that point, or the GP, they need to have skills to do it. It’s like your diabetes - diabetes should be managed by clinicians who have at least done some level of diabetes training.

Participant 11: Primary Care Practice Nurse with 21–30 years of experience.

There were multiple comments from participants about how they often felt that there is a “real complacency around asthma” (Participant 17: Integrated Care Specialist Nurse and Clinical Lead with over 30 years of experience) by many HCPs.

And people don’t see a diagnosis of asthma as being as serious as other conditions, because if we diagnosed a child with diabetes, we’d bring them into hospital for a week and work them up and have daily education.

Participant 9: Paediatric trainee doctor with a specialist interest in asthma and allergy (SPIN), with 11–20 years of experience

## Discussions

### Summary of findings

This study identified features for the development of clinical decision support system (CDSS) in asthma through opportunities for improving routine care. This knowledge should inform the direction of future development towards tools and systems which are desirable and useable in routine clinical practice. Although these findings may not be generalisable to all healthcare workers, they highlight some key and recurring topics. For example, the two most commonly discussed features which would be appreciated in a CDSS were medical history retrieval and best-practice reminders.

### Strengths and limitations

Past research has sought to identify facilitators and barriers to CDSS integration for asthma care, and the lack of collaboration with stakeholders early in development has been a recurring suggestion^34^. This study aimed to specifically explore the functions that were perceived as useful by healthcare providers, and through this we were able to identify precise targets for machine learning models, such as triaging patients for referral for biologic therapy.

One of the strengths of this study was the use of an inductive approach to qualitative analysis. Interview questions were designed to be open and non-directed, and data was analysed to capture as wide a range of suggested use cases as the cohort conceived.

Furthermore, the study was designed, and data analysis discussed, with a multi-disciplinary team of qualitative research experts, asthma CDSS designers, and HCPs. This allowed the questions and analysis to maximise utility and value to multiple stakeholders.

The primary limitations of this study were that the interviewer was not experienced in qualitative research, and it is inevitable that some bias will have been introduced during the interview stage, despite the exercises in reflexivity.

### Results in context

One of the highlighted functionalities desired in data driven support was the interpretation of pulmonary function tests, with one participant in particular expressing that they presumed AI could be used in the same way it has been integrated into routine radiology procedures^35^. Indeed there is great need for support in this area, with waiting times for many referrals having further lengthened since the COVID-19 pandemic, and research in test interpretation support has already demonstrated potential^21^. However, this comment also served as a reminder of the importance of learning from other disciplines in which data-driven support is more common and accepted. There are many parallels between facilitators and concerns highlighted during the early stages of AI radiology integration and the current study findings. For example, a Dutch study from 2020 highlighted the desirability of added value both clinically and operationally, the need for streamlined assimilation with other software, and the risk that benefits would be inconsistent if the integration of the system into existing workflows was not considered^36^. However, there are also marked differences between respiratory and radiology. For example, models developed for radiology are able to make use of highly standardised imaging data, whereas asthma care is far less standardised—in diagnosis, management, treatment, and data entry itself. Decisions in radiology are also typically binary (condition or status detected versus not), making the role of the AI model as an independent checker clearly defined. In contrast, asthma models often concern competing diagnoses, or probabilities of outcomes, which can make them harder to implement.

Our findings drew many parallels to a 2024 study specifically targeting paediatric asthma CDSSs in outpatient care^37^. A streamlined review of pertinent patient information was one of the most anticipated positive impacts on workflows, as well as promoting a shift from reactive to proactive care, and facilitating patient and parent education. Participants were generally supportive of risk prediction models, and in particular alerts notifying HCPs to follow-up with high-risk patients. However, regarding the specific mode of notifications, participants were varied in their responses. Some very wary of increasing the number of messages they received in their inbox, but equally there was concern that information only displayed in the electronic health record would not result in changes to care.

The primary difference in responses between primary and secondary care professionals was that secondary care respondents were often proposing solutions for use in primary care in order to reduce the number of unnecessary referrals into secondary care.

Any discussion about the design and implementation of CDSS should also consider what is feasible from a technological, financial, data governance, and compliance angle. The features participants discussed come with their own individual challenges in these fronts which would require extensive testing, piloting and validation. The functionality that poses the lowest data security and confidentiality risk is most likely medical history retrieval, as the platform could be set up without needing to send information outside of the host computer, and it would not directly influence clinical decisions.

Finally, we also note that we observed more similarity between the topics discussed by respondents from primary care providers than those from secondary care. In particular, there were more independent topics arising during the questions about their role and corresponding requirements—possibly owing the greater diversity of responsibilities in these respondents.

## Conclusions

This study underscores the potential of Clinical Decision Support Systems (CDSSs) to transform asthma care by addressing key challenges identified by healthcare providers. Through qualitative analysis, we highlighted specific areas—such as diagnosis support, medical history retrieval, and adherence to best practice guidelines—where CDSSs could significantly enhance clinical efficiency and patient outcomes. The integration of CDSSs into asthma care requires careful consideration of user needs, ensuring that tools are both usable and beneficial in routine practice. By prioritizing user-centred design and aligning with existing workflows, these systems can alleviate administrative burdens, streamline processes, and foster proactive patient management.

Our findings align with broader trends in healthcare, where data-driven solutions are increasingly leveraged to optimize clinical decision-making. Parallels drawn with successful CDSS applications in other medical fields, such as radiology, highlight the importance of interdisciplinary learning and adaptation. Future development should focus on creating intuitive, reliable systems that enhance rather than disrupt existing practices, thus ensuring widespread adoption and sustained impact.

## Data Availability

The datasets (interview transcripts) generated and analysed during the current study are not publicly available due to risk of re-identification, but are available from the corresponding author on reasonable request.

## Abbreviations

A&E: Accident and Emergency department
AI: Artificial intelligence
ARRS: Additional Roles Reimbursement Scheme
BTS: British Thoracic Society
CDSS: Clinical decision support system
COPD: Chronic obstructive pulmonary disease
GP: General practitioner
HCP: Healthcare provider
ICS: Inhaled corticosteroids
NICE: The National Institute for Health and Care Excellence
SABA: Short-acting beta-2 agonist
SIGN: Scottish Intercollegiate Guidelines Network
UK: United Kingdom
